# Combined cartilage thickness and mechanical property mismatch drives local strain amplification at the patellar osteochondral allograft interface

**DOI:** 10.1007/s10237-026-02128-9

**Published:** 2026-09-12

**Authors:** Michael A. Hernández Lamberty, John A. Grant, Ellen M. Arruda, Rhima M. Coleman

**Affiliations:** 1 Department of Mechanical Engineering, University of Michigan, Ann Arbor, MI USA; 2 Department of Biomedical Engineering, University of Michigan, Ann Arbor, MI USA; 3 Program in Macromolecular Science and Engineering, University of Michigan, Ann Arbor, MI USA; 4 Department of Orthopaedic Surgery, University of Michigan, Ann Arbor, MI USA

**Keywords:** Finite element analysis, Cartilage, Osteochondral allograft transplant, Patella, Patellofemoral joint

## Abstract

Patellar osteochondral allograft (OCA) transplantation treats focal cartilage defects, but donor-recipient cartilage thickness and mechanical compatibility are not routinely quantified during graft selection. This idealized computational study evaluated associations of donor-to-recipient (*D*/*R*) thickness ratio, absolute stiffness, stiffness contrast, and depth-dependent stiffness with interface-local strain. Two-dimensional axisymmetric finite element models were subjected to a spatially uniform nominal pressure of 1.00 MPa. Nine *D*/*R* ratios (0.33–3.25) and eleven homogeneous donor/recipient (DC/RC) Young’s modulus combinations (2.50-7.00 MPa) yielded 99 homogeneous cases; nine functionally graded material (FGM) cases were evaluated. Primary outcomes were 95th percentile (P95) values of the nodewise compressive strain magnitude and tensor maximum shear strain within a fixed 0.25-mm local interface region of interest (ROI). This robust upper-tail measure is less sensitive than a single nodal maximum. In the matched homogeneous control, P95 values were 0.031 and 0.015. The largest homogeneous values were 0.412 and 0.347 at *D*/*R*=3.25 and DC/RC=7.0/2.5 MPa. Same-side concentration factors normalized local P95 to median strain in the same model’s 0.75-1.00 mm far-field band. Pooled factors were 3.71 ± 2.04 for compression and 6.03 ± 3.79 for maximum shear in homogeneous cases (*n* = 198 model-sides), and 3.51 ± 2.17 and 5.69 ± 4.03 in FGM cases (*n* = 18). FGM values were lower than both extreme homogeneous mismatch references at every *D*/*R* ratio. Across formulations, thickness disparity remained associated with interface-local upper-tail strain. These results support evaluating donor-recipient cartilage thickness and material compatibility alongside surface congruity and osseous integration in future experimental and patient-specific modeling studies.

**Supplementary Information:**

The online version contains supplementary material available at https://doi.org/10.1007/s10237-026-02128-9.

## Introduction

Articular cartilage is a specialized soft tissue found at the end of the bones of diarthrodial joints. It provides a near-frictionless surface for the load transfer between bones (Sophia Fox et al. 2009). This mechanical function is achieved through its complex hierarchical structure composed of a depth-dependent collagen fibril network. Overall, the articular cartilage is composed of an extracellular matrix (ECM) that consists primarily of water, collagen, and proteoglycans. Proteoglycans contain negatively charged glycosaminoglycan side chains that attract mobile ions and water into the tissue. The resulting influx of fluid generates osmotic swelling pressure that is resisted by the tensile stiffness of the collagen fibril network which enables articular cartilage to withstand compressive loads (Soltz and Ateshian 1998; Huang et al. 2001). Variations in the collagen fiber orientation, and proteoglycan and chondrocyte concentrations across the tissue depth give rise to the superficial, middle and deep zones of the articular cartilage, each with distinct structural organization and mechanical properties (Buckwalter et al. 1994; Antons et al. 2018).

Cartilage injuries are observed in up to 66% of patients undergoing knee arthroscopy procedures (Curl et al. 1997; Årøen et al. 2004). Full-thickness chondral injuries are observed in 4.6-6.2% of all patients, but the prevalence of full-thickness injuries increases to 36% in athletes undergoing knee arthroscopy (Flanigan et al. 2010). Among all the locations of chondral injuries that are found in the knee, approximately 20% are located in the patella (Curl et al. 1997). Cartilage has limited regenerative capacity due to its being both aneural and avascular. This often means that surgical methods are necessary to repair full-thickness injuries of the articular cartilage. If these chondral injuries are not addressed, they may compromise the joint function and accelerate the onset of osteoarthritis (Bhosale and Richardson 2008).

Osteochondral allograft (OCA) transplantation is the standard procedure for treating articular cartilage defects greater than 2 cm^2^ (Hevesi et al. 2021). The OCA transplant includes a cylindrical graft of healthy cartilage and the subchondral bone that is then press-fit into the recipient’s prepared defect site (Williams et al. 2004; Montgomery et al. 2014). One of the main benefits of the OCA procedure is that in a single-stage operation, the cartilage surface is restored. OCA has gained popularity among athletes with full-thickness defects due to its faster rehabilitation times and, consequently, faster return to play (Krych et al. 2016). Despite the advantages, OCA procedures are not without drawbacks. Previous work done by Familiari et al. showed that OCAs fail at a rate of 13% at 5 years, 21% at 10 years, 27% at 15 years, and 33% at 20 years following the procedure (Familiari et al. 2018). Patellar OCA transplant procedures have also shown high rates of failure and reoperation long term; at 15 years following the procedure, up to 20% of grafts failed, and 52% of the patients required reoperation (Chahla et al. 2019). These outcomes emphasize the importance of identifying mechanical factors that may affect the graft environment while recognizing that clinical outcome is multifactorial.

The primary parameters for successful OCA transplant procedures are articular cartilage congruity and osseous integration (Lai et al. 2022). However, both biological and mechanical factors influence the success of the procedure. In the patellofemoral joint, residual maltracking of the patella, joint instability, or incongruent subchondral surfaces can exacerbate the risk of OCA failure (Haber et al. 2019; Ackermann et al. 2019; Patel et al. 2021). Although the consequences of graft elevation and subchondral surface incongruity have been studied and are actively managed clinically, cartilage thickness mismatch between the donor and recipient tissue is not currently considered in graft-selection protocols.

Our group previously showed that donor-to-recipient (*D*/*R*) cartilage thickness disparity in patellar OCA models produced local stress concentrations near the graft boundary, with stresses reaching approximately twice the applied surface pressure (Rosario et al. 2023). Those stress-based findings motivated the present evaluation of whether thickness and material-property variation are also associated with interface-local compressive and maximum shear strain. Because stress and strain describe distinct aspects of the mechanical response, the current strain outcomes were evaluated directly rather than inferred from the prior stress analysis. Previous in vitro studies using cyclic compressive loading reported reduced chondrocyte viability at applied stresses of ~6 MPa and above, with more severe loading procedure producing greater cell loss and matrix damage (Clements et al. 2001; Thibault et al. 2002). Other work using articular cartilage explants demonstrated that compressive strains exceeding ~18% can induce chondrocyte apoptosis in the deep zone of the cartilage (Bartell et al. 2015; Huang et al. 2020). Notably, patellofemoral joint loading varies substantially across daily and athletic activities (Huberti and Hayes 1984; Lenhart et al. 2015). Because experimental injury thresholds depend strongly on loading protocol, rate, duration, tissue depth, and strain definition, it is difficult to identify a single physiological cartilage death threshold. Nevertheless, localized strain amplification at the donor-recipient interface could expose a small region of cartilage to a mechanical environment that is distinct from the surrounding tissue, providing a plausible pathway by which mechanical mismatch may contribute to progressive degeneration and graft failure.

In addition to geometric mismatches, mechanical property mismatch between the donor and recipient cartilage might contribute to altered graft mechanics. Articular cartilage is an inhomogeneous tissue that has spatially varying mechanical properties (Deneweth et al. 2013, 2015; Antons et al. 2018). Prior studies have shown that the material constants for linear elastic, biphasic, or hyperelastic cartilage material models vary across joints and individuals (Armstrong and Mow 1982; Pierce et al. 2009; Antons et al. 2018). As a result, donor cartilage used for OCA transplantation may have a different stiffness than the surrounding recipient cartilage, even when the graft is geometrically well matched. This mechanical property disparity may alter load transfer across the donor-recipient cartilage interface. Therefore, the goal of this study was to identify the combined effect of *D*/*R* cartilage thickness and mechanical property mismatches on local strain amplification in a human patella OCA finite element (FE) model. We hypothesized that a larger disparity in *D*/*R* cartilage thickness, particularly when compounded by a mechanical property mismatch, would increase local compressive and maximum shear strain within the interface.

## Methods

### Finite element model

#### Model geometry

Simplified two-dimensional axisymmetric FE models of the patella cartilage with an osteochondral allograft (OCA) were created in ABAQUS 2022 (SIMULIA) to understand the effects of donor-recipient cartilage thickness and mechanical property mismatch. The model dimensions and the *D*/*R* thickness ratios were informed by nano-computed tomography measurements of cadaveric patellar OCA transplants performed by a sports medicine orthopedic surgeon (Patel et al. 2021), for which image acquisition and segmentation were described previously (Rosario et al. 2023). The models represent an idealized local cartilage interface and were not intended to reproduce patient-specific joint contact or predict clinical outcome.

**Fig. 1:**
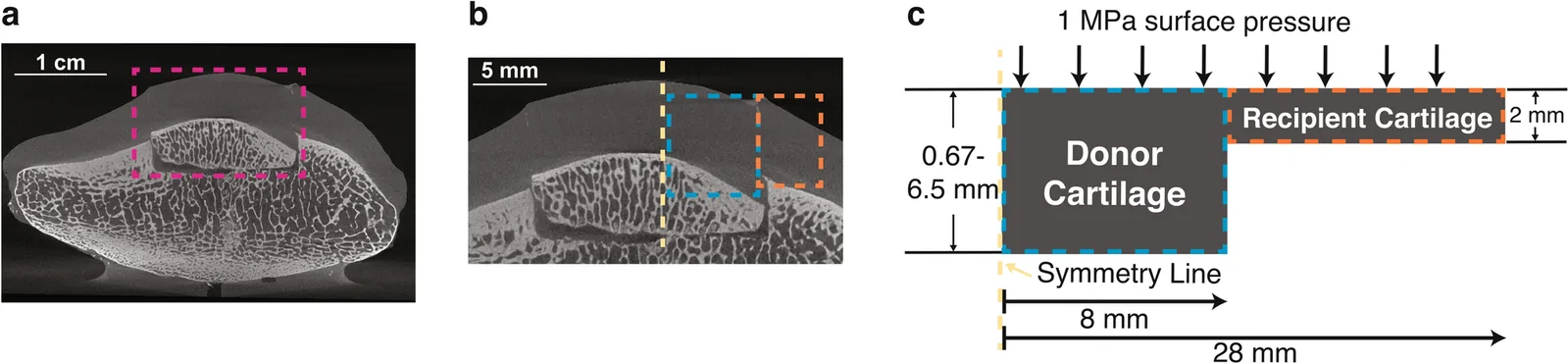
**a** Representative nano-CT image of patella following a successful OCA procedure. In this image, cartilage thickness mismatches can be observed on both sides of the OCA graft. **b** Enlarged donor–recipient interface region used to identify the donor and recipient cartilage domains and the axisymmetric modeling plane. The yellow line represents the line of symmetry for the model, the cyan box represents the donor cartilage section, and the orange box represents the recipient cartilage section. **c** Simplified two-dimensional axisymmetric model with donor cartilage (DC), recipient cartilage (RC), the donor–recipient interface at *x* = 8 mm, and a spatially uniform nominal surface pressure of 1.00 MPa

Using the characteristics of the scans, the donor cartilage was modeled with a radius of 8 mm, while the recipient cartilage had a radius of 28 mm. The donor-recipient junction was represented using a rounded transition generated with a 20 µm fillet radius and 10 µm side dimensions on the donor and recipient sides of the centered material boundary. In the final model implementation, half of the fillet transition was assigned to donor cartilage, and half was assigned to recipient cartilage so that the material boundary remained centered at the graft interface. Cartilage thickness in the models was varied by changing the donor cartilage thickness while keeping the recipient cartilage thickness at 2 mm, resulting in nine *D*/*R* ratios: 0.33, 0.50, 0.67, 0.80, 1.00, 1.25, 1.50, 2.00, and 3.25 (Fig. 1; Table 1). Thus, *D*/*R* < 1 denotes recipient-side-thicker configurations, *D*/*R* = 1 denotes matched thickness, and *D*/*R* > 1 denotes donor-side-thicker configurations. Comparisons of chamfer, and fillet interface regularization are reported in Supplemental Information, Appendix C.

**Table 1:** Donor-to-recipient cartilage thickness ratios evaluated in the finite element models

Configuration	*D*/*R* thickness ratios	Number
Recipient-side thicker (*D*/*R* < 1)	0.33, 0.50, 0.67, 0.80	4
Thickness matched (*D*/*R* = 1)	1.00	1
Donor-side thicker (*D*/*R* > 1)	1.25, 1.50, 2.00, 3.25	4

Nine donor-to-recipient (*D*/*R*) cartilage thickness ratios were modeled to evaluate the effect of geometric mismatch on local-interface 95th percentile strain outcomes. Recipient cartilage thickness was held at 2 mm, and donor cartilage thickness was varied to produce the listed *D*/*R* ratios

#### Mesh generation

The FE models were discretized using 8-node biquadratic axisymmetric elements (CAX8). A quadratic mesh was generated with an average element edge length of approximately 50 μm throughout the whole cartilage. To increase the accuracy of the local strain gradients near the donor-recipient interface, the mesh was progressively refined within 2 mm of the graft boundary. The refinement linearly decreased the edge length of the quadratic elements from 50 μm to 5 μm at the interface. This refinement method was chosen to capture the localized HSR observed near the graft boundary. A mesh convergence analysis was performed by progressively refining the mesh near the graft boundary. Changes in the primary strain outcomes between successive mesh refinements were less than 3%, indicating convergence while avoiding a disproportionate increase in computation time.

#### Material models

##### Homogeneous linear elastic models

In the initial set of FE analyses, the articular cartilage was modeled as a homogeneous isotropic linear elastic material. Poisson’s ratio of 0.46 was used for all models (Jin and Lewis 2004). The stiffness or Young’s modulus was varied between 2.5 MPa and 7.0 MPa to represent reported ranges of human articular cartilage stiffness under physiological loading conditions and at various locations through the thickness of the cartilage (Chen et al. 2001; Antons et al. 2018).

Mechanical property mismatches were introduced by independently assigning Young’s modulus values to the donor cartilage (DC) and recipient cartilage (RC) regions. For each *D*/*R* ratio, either the DC or RC modulus was held at 7.0 MPa while the other region was assigned 6.0, 5.0, 4.0, 3.0, or 2.5 MPa; the matched 7.0/7.0 MPa condition was also included. This produced eleven DC/RC modulus combinations per thickness ratio (Table 2). A thickness-matched and mechanically matched model (*D*/*R* = 1; DC/RC = 7.0/7.0 MPa) served as the control condition.

**Table 2:** Donor/recipient Young’s modulus combinations evaluated in the homogeneous linear elastic models

Configuration	DC/RC Young’s modulus (MPa/MPa)	Number
Stiffness matched	7.0/7.0	1
Donor stiffer	7.0/6.0, 7.0/5.0, 7.0/4.0, 7.0/3.0, 7.0/2.5	5
Recipient stiffer	6.0/7.0, 5.0/7.0, 4.0/7.0, 3.0/7.0, 2.5/7.0	5

Eleven donor/recipient (DC/RC) Young’s Modulus combinations were evaluated for each *D*/*R* thickness ratio: one matched 7.0/7.0 MPa condition, five conditions with donor cartilage fixed at 7.0 MPa, and five conditions with recipient cartilage fixed at 7.0 MPa

##### Functionally graded material models

**Fig. 2:**
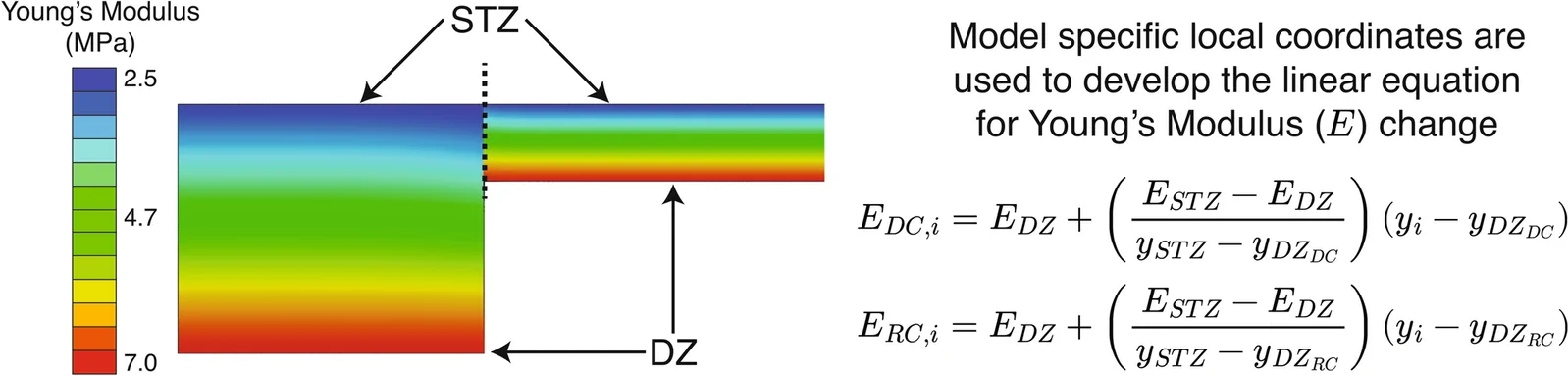
A functionally graded material (FGM) model was built in ABAQUS using a user-defined field (USDFLD) to define changing material properties along the thickness of the articular cartilage using literature-reported values. Young's modulus was varied linearly through cartilage depth from 2.5 MPa (${E}_{\mathrm{S}\mathrm{T}\mathrm{Z}}$) at the superficial tangential zone (STZ) to 7.0 MPa (${E}_{\mathrm{D}\mathrm{Z}}$) at the deep zone (DZ). Donor and recipient cartilage were assigned region-specific coordinate-based modulus fields using model coordinates and field-variable-dependent elastic material definitions. The equations show the linear interpolation used to assign the donor cartilage modulus (${E}_{\mathrm{D}\mathrm{C},i}$) and recipient cartilage modulus (${E}_{\mathrm{R}\mathrm{C},i}$) based on the local through-thickness coordinate ${y}_{i}$. The contour illustrates a representative continuous depth-dependent stiffness field; visible color bands are post-processing intervals, not manually assigned discrete material layers

To represent depth-dependent mechanical behavior of articular cartilage, an additional set of FE models incorporated a functionally graded material (FGM) formulation. A user-defined field subroutine (USDFLD) was implemented in ABAQUS to assign spatially varying Young’s modulus values as a function of the through-thickness coordinates of the cartilage. The modulus was prescribed to vary linearly from a starting value of 2.5 MPa (${E}_{\mathrm{S}\mathrm{T}\mathrm{Z}}$) at the superficial tangential zone (STZ) of the cartilage to a value of 7.0 MPa (${E}_{\mathrm{D}\mathrm{Z}}$) at the deep zone (DZ) (Fig. 2). These values are consistent with previous experimental zonal variations in cartilage stiffness (Töyräs et al. 2001; Jin and Lewis 2004; Antons et al. 2018). The field variable was evaluated at material integration points, and ABAQUS interpolated the elastic modulus from the corresponding field-variable material using the following equations:

$${E}_{\mathrm{D}\mathrm{C},i}={E}_{\mathrm{D}\mathrm{Z}}+\left(\frac{{E}_{\mathrm{S}\mathrm{T}\mathrm{Z}}-{E}_{\mathrm{D}\mathrm{Z}}}{{y}_{{\mathrm{S}\mathrm{T}\mathrm{Z}}_{\mathrm{D}\mathrm{C}}}-{y}_{{\mathrm{D}\mathrm{Z}}_{\mathrm{D}\mathrm{C}}}}\right)\left({y}_{i}-{y}_{{\mathrm{D}\mathrm{Z}}_{\mathrm{D}\mathrm{C}}}\right)$$

$${E}_{\mathrm{R}\mathrm{C},i}={E}_{\mathrm{D}\mathrm{Z}}+\left(\frac{{E}_{\mathrm{S}\mathrm{T}\mathrm{Z}}-{E}_{\mathrm{D}\mathrm{Z}}}{{y}_{{\mathrm{S}\mathrm{T}\mathrm{Z}}_{\mathrm{R}\mathrm{C}}}-{y}_{{\mathrm{D}\mathrm{Z}}_{\mathrm{R}\mathrm{C}}}}\right)\left({y}_{i}-{y}_{{\mathrm{D}\mathrm{Z}}_{\mathrm{R}\mathrm{C}}}\right)$$

where ${y}_{i}$ is the material-integration-point through-thickness coordinate, ${y}_{{\mathrm{D}\mathrm{Z}}_{\mathrm{D}\mathrm{C}}}$ and ${y}_{{\mathrm{S}\mathrm{T}\mathrm{Z}}_{\mathrm{D}\mathrm{C}}}$ are the donor DZ and STZ coordinate, and ${y}_{{\mathrm{D}\mathrm{Z}}_{\mathrm{R}\mathrm{C}}}$ and ${y}_{{\mathrm{S}\mathrm{T}\mathrm{Z}}_{\mathrm{R}\mathrm{C}}}$ are the corresponding recipient coordinates. The coordinates were defined separately for each geometry so that the full 2.5-7.0 MPa gradient was normalized to the local donor or recipient thickness. Nine FGM cases, one for each *D*/*R* ratio, were analyzed. All other modeling assumptions, including geometry, thickness ratio, mesh density, boundary conditions, and loading, were identical to those used in the homogeneous material model.

#### Boundary conditions and loading

Translation of nodes along the subchondral surface was fixed, and axisymmetric boundary conditions were applied along the central axis. The main homogeneous and FGM datasets used a conforming continuous shared-node mesh across the donor-recipient cartilage boundary; therefore, displacement compatibility was inherent and no tied constraint, separation, or sliding was permitted. This interface represents an idealized integrated condition. Geometric nonlinearity was enabled in the static compression step. A spatially uniform nominal pressure of 1.00 MPa was prescribed normally to the cartilage surface over a 26 mm radius, leaving a 2 mm unloaded edge buffer to reduce edge effects and excessive element distortion. A qualitative whole-model assessment of the applied supports, deformed configuration, U2 field, and separation of the donor-recipient interface from the unloaded outer-edge buffer is provided in Supplemental Information, Appendix A. The pressure served as a controlled distributed load for comparison across model configurations and was not intended to reproduce spatially heterogeneous in vivo patellofemoral contact pressures.

### Study design and data analysis

Two parametric studies were conducted. In the first study, donor-recipient cartilage thickness ratio and mechanical property mismatch were varied using the homogeneous linear elastic cartilage model described in Sect. 2.1.3.1. Nine *D*/*R* thickness ratios ranging from 0.33 to 3.25 were combined with eleven donor/recipient Young’s modulus combinations. In the second study, depth-dependent material behavior was evaluated using the FGM formulation described in Sect. 2.1.3.2. The same set of *D*/*R* thickness ratios was evaluated in the FGM study to assess whether a more physiologically representative through-thickness stiffness gradient altered the local strain response to cartilage thickness mismatch. Separate-body contact sensitivity models required a rigid-body representation of the underlying bone and surface-to-surface cartilage-cartilage and cartilage–bone interactions because the primary shared-node support formulation could not be applied consistently after the bodies were separated, particularly at *D*/*R* ≠ 1 (Supplemental Information, Appendix F).

**Fig. 3:**
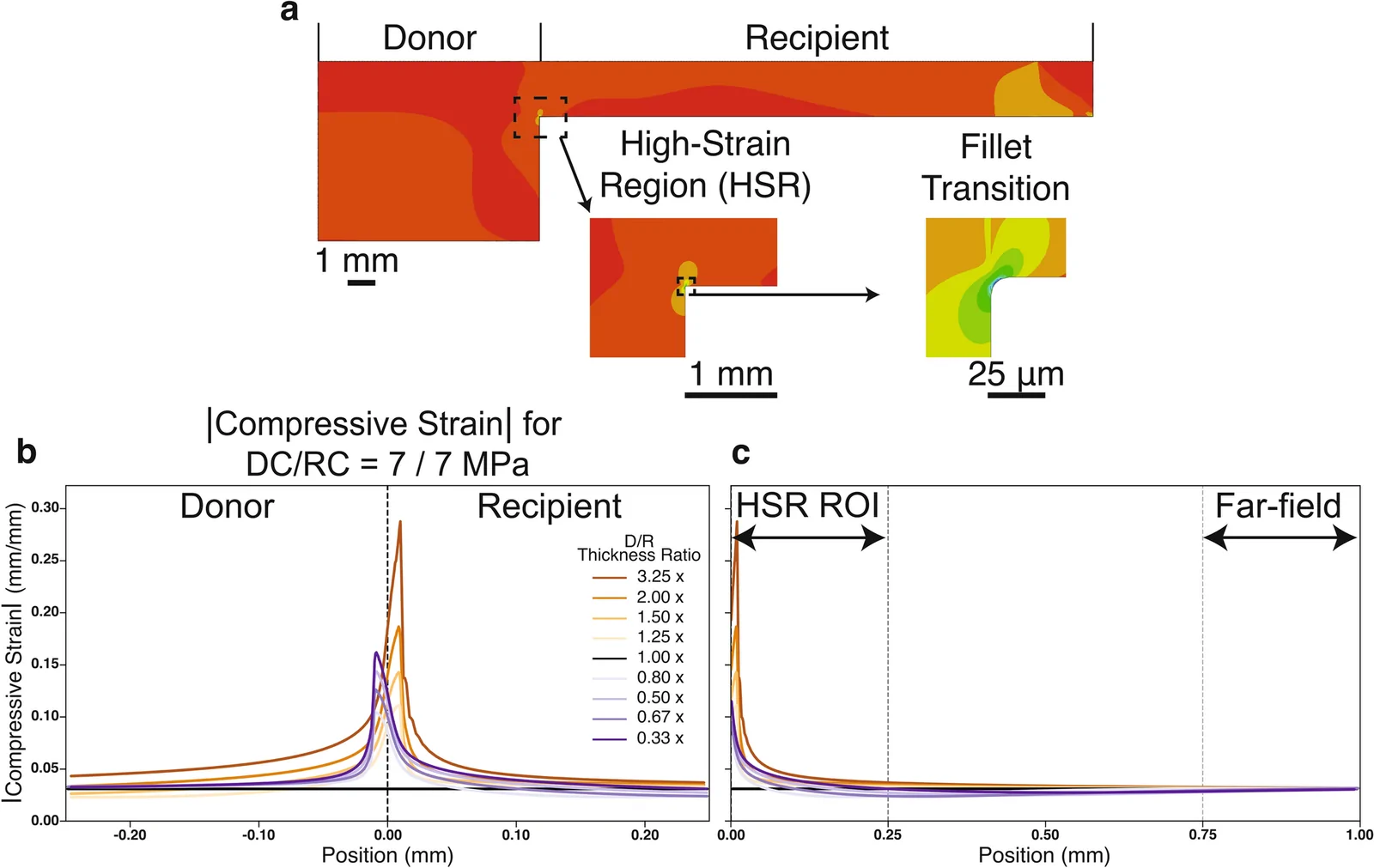
**a** Compressive-strain-magnitude contour showing a threshold-defined high strain region (HSR) near the donor-recipient interface and rounded fillet. **b** Pointwise compressive-strain-magnitude profiles for homogeneous DC/RC = 7.0/7.0 MPa models across *D*/*R* ratios, with position reported relative to the interface **c** Analysis-region schematic showing the local HSR region of interest (ROI) used for the primary 95th percentile values, 0 to 0.25 mm from the interface, and the far-field normalization band, 0.75 to 1.0 mm from the interface. Exact interface nodes were excluded before calculating the 95th percentile value

Logarithmic strain ($\mathrm{L}\mathrm{E}$) was obtained from the final frame of the compression step. The extraction scripts selected undeformed mesh nodes along an interface-centered path from *x* = 7 to 9 mm, with the donor-recipient boundary at *x* = 8 mm (Fig. 3b) which is where the HSR was observed (Fig. 3a). The $\mathrm{L}\mathrm{E}$ field was requested at the ABAQUS element nodal positions along the path. Each position contains one value for each element-node pair; when $\mathrm{L}\mathrm{E}$ was stored at integration points, ABAQUS extrapolated the various strain components (${\mathrm{L}\mathrm{E}}_{11}$, ${\mathrm{L}\mathrm{E}}_{22}$, ${\mathrm{L}\mathrm{E}}_{33}$, and ${\mathrm{L}\mathrm{E}}_{12}$) to the nodes of each contributing element. The scripts then formed one nodewise strain tensor by taking an unweighted componentwise mean across connected elements. Averaging was performed separately within the donor- and recipient-cartilage element sets, preserving side-specific strain estimates at nodes shared by the two regions. Components were averaged before principal strains were calculated.

ABAQUS reports ${\mathrm{L}\mathrm{E}}_{12}$ as an engineering logarithmic shear component; therefore, the following equations were used to calculate the tensor off-diagonal components and in-plain principal logarithmic strains:

$${\varepsilon}_{12}=\frac{{\mathrm{L}\mathrm{E}}_{12}}{2}$$

$${\varepsilon}_{\pm }=\frac{{\mathrm{L}\mathrm{E}}_{11}+{\mathrm{L}\mathrm{E}}_{22}}{2}\pm \sqrt{{\left(\frac{{\mathrm{L}\mathrm{E}}_{11}-{\mathrm{L}\mathrm{E}}_{22}}{2}\right)}^{2}+{\varepsilon}_{12}^{2}}$$

$${\varepsilon}_{\mathrm{m}\mathrm{i}\mathrm{n}}=\mathrm{min}\left({\varepsilon}_{-},{\mathrm{L}\mathrm{E}}_{33}\right),{\varepsilon}_{\mathrm{m}\mathrm{a}\mathrm{x}}=\mathrm{max}\left({\varepsilon}_{+},{\mathrm{L}\mathrm{E}}_{33}\right)$$

$${\varepsilon}_{\mathrm{s}\mathrm{h}\mathrm{e}\mathrm{a}\mathrm{r},\mathrm{m}\mathrm{a}\mathrm{x}}=\frac{{\varepsilon}_{\mathrm{m}\mathrm{a}\mathrm{x}}-{\varepsilon}_{\mathrm{m}\mathrm{i}\mathrm{n}}}{2}$$

where ${\varepsilon}_{\mathrm{m}\mathrm{i}\mathrm{n}}$ and ${\varepsilon}_{\mathrm{m}\mathrm{a}\mathrm{x}}$ are the minimum and maximum principal logarithmic strain and ${\varepsilon}_{\mathrm{s}\mathrm{h}\mathrm{e}\mathrm{a}\mathrm{r},\mathrm{m}\mathrm{a}\mathrm{x}}$ is the maximum shear strain. Exact interface nodes at *x* = 8 mm were excluded before percentile calculations. The primary strain outcomes were compressive strain magnitude, calculated from the magnitude of ${\varepsilon}_{\mathrm{m}\mathrm{i}\mathrm{n}}$, and ${\varepsilon}_{\mathrm{s}\mathrm{h}\mathrm{e}\mathrm{a}\mathrm{r},\mathrm{m}\mathrm{a}\mathrm{x}}$. 95th percentile strain values were calculated from nodal strain within the local interface region of interest (ROI) on each side of the interface. The full extracted path was used to define the local interface ROI, transition band, and far-field normalization band (Fig. 3c). For the FGM, 95th percentile strain outcomes were compared directly to the extreme and control cases from the homogeneous material simulation to assess the influence of depth-dependent material behavior.

### High-strain-region extent and local/far-field concentration factors

High-strain-region (HSR) extent and the fixed analysis regions served different purposes. The analysis regions were applied consistently across all models: The local interface ROI was defined as $0<\left|x-8 mm\right|\le 0.25$ mm, the transition band was defined as $0.25<\left|x-8 mm\right|<0.75$ mm, and the far-field normalization band was defined as $0.75\le \left|x-8 mm\right|\le 1.00$ mm. The local ROI defined the nodes used to calculate side-specific 95th percentile strain, whereas the far-field band defined the same-side median strain used for normalization. HSR extent was evaluated separately on the donor and recipient sides as a containment check and did not determine the ROI width. For each strain measure, HSR extent was defined as the farthest distance from the interface at which strain met or exceeded a selected diagnostic threshold. A strain value of 0.18 was used as the primary diagnostic threshold. These thresholds were used only to evaluate the spatial extent of the localized high-strain region and were not treated as validated biological or clinical injury thresholds. The HSR counts and maximum extents at the primary 0.18 threshold are reported in Supplemental Information Appendix B.

For each model, metric and side of interface, a dimensionless concentration factor was calculated as:

$${K}_{95,m,s}=\frac{P95\left({{\varepsilon}_{m,s}}_{Local ROI}\right)}{median ({{\varepsilon}_{m,s}}_{far-field})}$$

where m denotes either compressive strain magnitude or maximum shear strain, s denotes the donor or recipient side, $P95\left({{\varepsilon}_{m,s}}_{Local ROI}\right)$ is the 95th percentile strain within the local interface ROI, and $median ({{\varepsilon}_{m,s}}_{far-field})$ is the median strain in the same-side far-field band. The far-field median value was used as the denominator because the far-field band was intended to represent the typical background strain state away from the interfaces, whereas the $P95\left({{\varepsilon}_{m,s}}_{Local ROI}\right)$ summarize the robust upper-tail strain response within the HSR. The concentration factor was calculated separately for the donor and recipient side of each model. The resulting side-specific concentration factors were summarized as the mean ± one standard deviation.

## Results

### Local 95th percentile strain across cartilage thickness and stiffness conditions

**Fig. 4:**
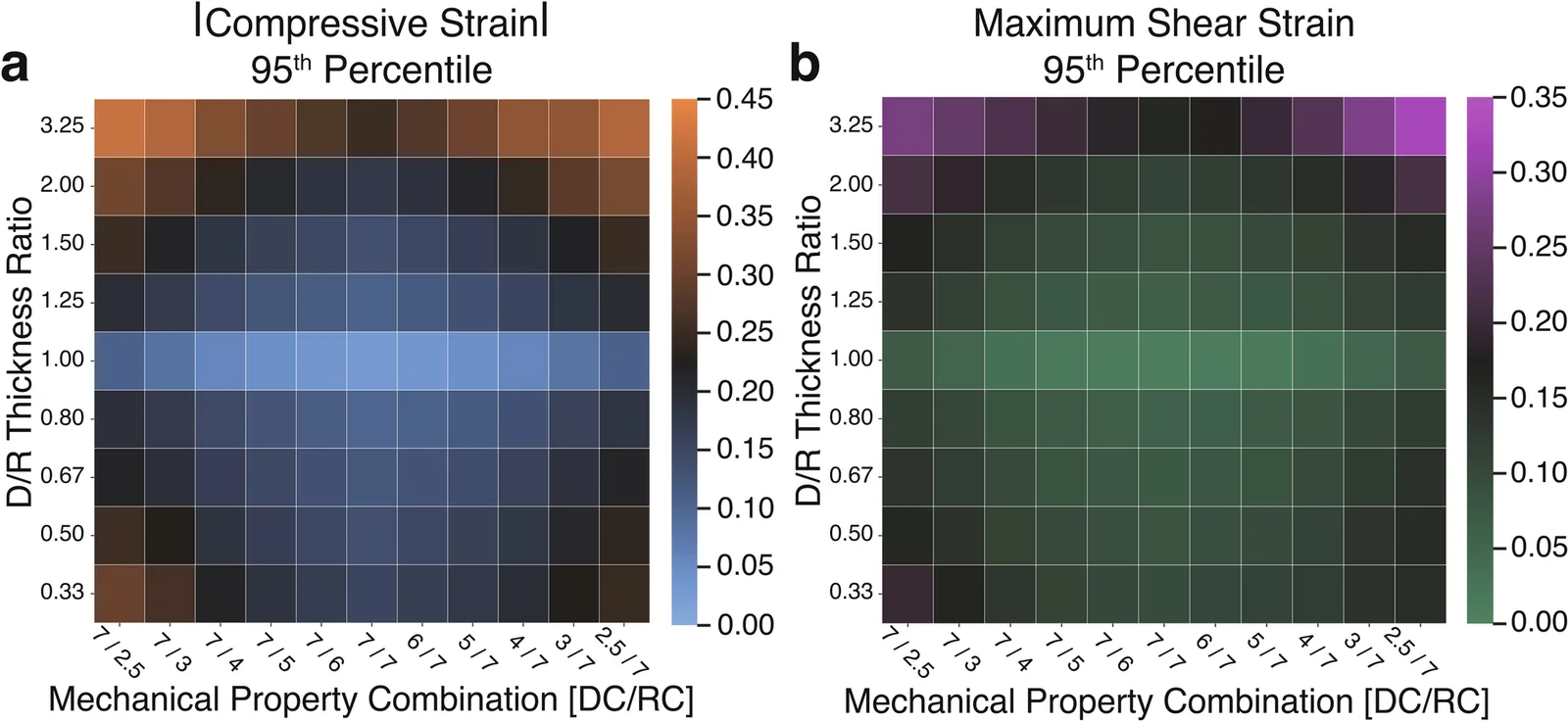
Local-interface-ROI 95th percentile in homogeneous linear elastic models. **a** Compressive strain magnitude and **b** maximum shear strain across the nine *D*/*R* thickness ratios and eleven donor/recipient modulus combinations (DC/RC, MPa). Each heatmap entry is the 95th percentile of nodal strain values within the local interface ROI on the side with the larger local-ROI response after excluding exact interface nodes. Values are dimensionless strain (mm/mm). These heatmaps summarize localized upper-tail interface strain and do not use the full 0 to 1 mm path as the percentile population

Homogeneous linear elastic axisymmetric finite element models demonstrated that donor-recipient cartilage thickness mismatch produced localized upper-tail strain amplification at the graft boundary. In the thickness- and modulus-matched control model (*D*/*R* = 1, donor/recipient modulus = 7.0/7.0 MPa), local-interface-ROI 95th percentile values were 0.031 for compressive strain magnitude (Fig. 4a) and 0.015 for maximum shear strain (Fig. 4b). Introducing mechanical property mismatch while maintaining matched thickness (*D*/*R* = 1) increased local 95th strain on the side of the large local response. For the thickness-matched model, the 7.0/2.5 MPa donor/recipient modulus case increased the 95th percentile compressive and maximum shear strain to 0.107 and 0.0679, respectively, representing a 245% increase in compressive strain magnitude relative to the control model. The reciprocal 2.5/7.0 MPa case produced similar values of 0.106 for the compressive strain and 0.0668 for the maximum shear strain, representing a 242% increase in compressive strain from the control model due to the change in mechanical properties in the donor cartilage.

**Fig. 5:**
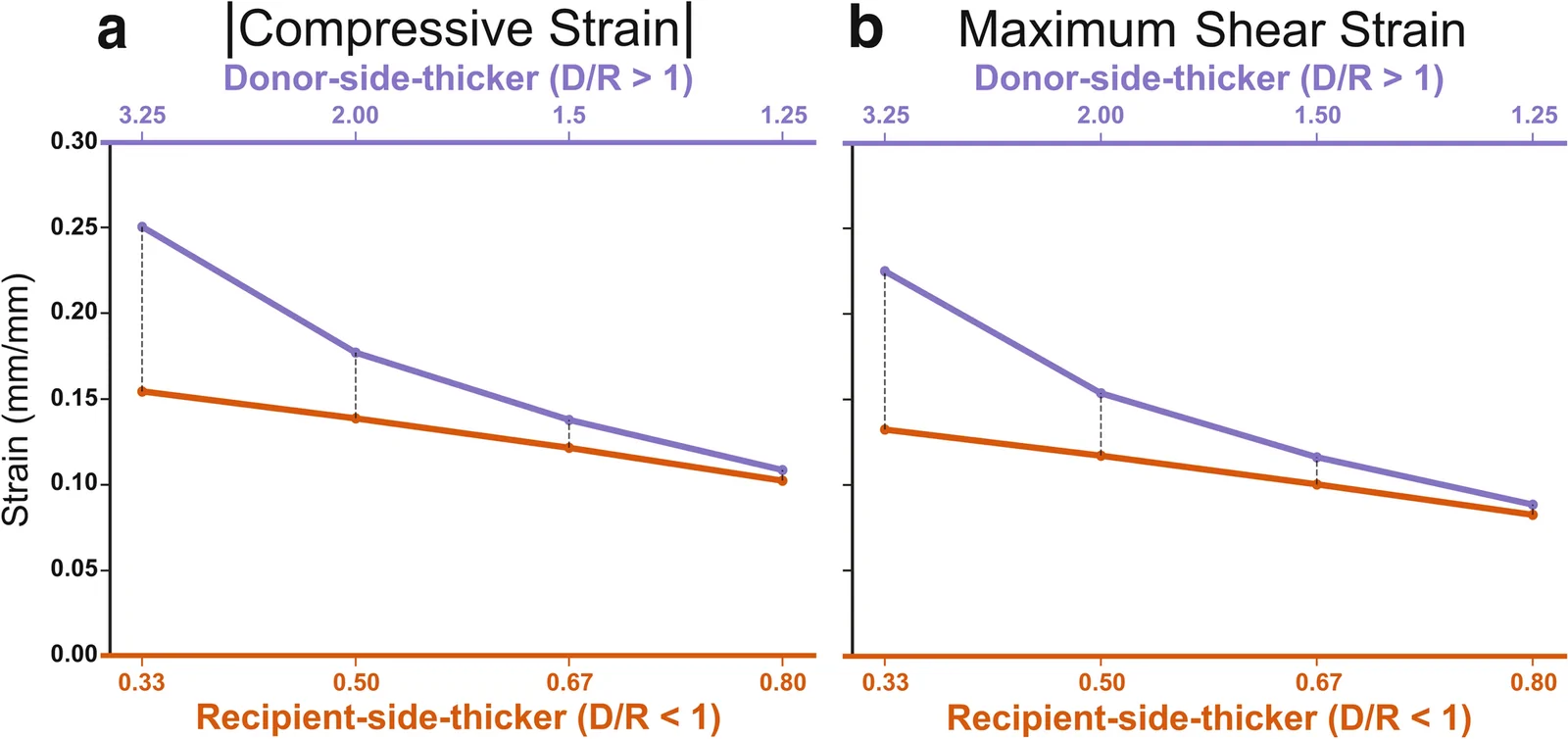
Local-interface-ROI 95th percentile strain for paired opposing thickness-disparity configurations in homogeneous matched-stiffness models (DC/RC = 7.0/7.0 MPa). The larger side-specific 95th percentile value is shown for **a** compressive strain magnitude and **b** tensor maximum shear strain. Donor-side-thicker configurations (*D*/*R* > 1; purple, upper axis) are paired with recipient-side-thicker configurations (*D*/*R* < 1; orange, lower axis)

Varying *D*/*R* cartilage thickness ratio while maintaining matched mechanical properties (7.0/7.0 MPa) also produced substantial 95th percentile strain amplification for both compressive and shear strains. Increasing *D*/*R* from 1 to 3.25 increased the local 95th percentile compressive strain from 0.031 to 0.250 and maximum shear strain from 0.015 to 0.225. Decreasing *D*/*R* from 1 to 0.33 increased these values to 0.154 and 0.132, respectively. These results demonstrate an increase of 706% for the *D*/*R* of 3.25 and 397% for the *D*/*R* of 0.33. Across paired opposing mismatch direction, donor-side-thicker configurations (*D*/*R* > 1) produced higher local 95th percentile strain than recipient-side-thicker configurations (*D*/*R* <1). The difference narrowed as *D*/*R* approached 1 (Fig. 5).

The largest absolute homogeneous 95th percentile strain values occurred when extreme thickness and stiffness were combined. At *D*/*R* = 3.25 and DC/RC = 2.5/7.0 MPa, compressive and maximum shear strains were 0.386 and 0.344, respectively; the DC/RC = 7.0/2.5 MPa assignment at the same thickness ratio produced 0.412 and 0.347. These results identify the combined configuration producing the largest values in the tested matrix, but do not isolate stiffness mismatch from the effect of absolute stiffness and geometry-dependent load sharing.

### Local/far-field concentration factors

The 0.25-mm local ROI was used consistently across all models to calculate side-specific 95th percentile strain, while the 0.75-1.00-mm band was used to characterize far-field strain. The HSR-extent analysis was used as a separate containment check and did not determine the ROI width. At the diagnostic threshold of 0.18, the farthest threshold-exceeding node in the homogeneous models was 0.0218 mm from the interface for compression and 0.0138 mm for maximum shear. The corresponding maximum distances in the FGM models were 0.0125 and 0.0112 mm, respectively. Thus, every HSR identified at the 0.18 threshold was contained well within the fixed 0.25-mm local ROI.

Same-side concentration factors indicated that local upper-tail strain was elevated relative to each model’s background strain (Table 3). Across 99 homogeneous configurations pooled over both sides (*n* = 198 model-sides), the mean concentration factors were 3.71 ± 2.04 for compressive strain magnitude and 6.03 ± 3.79 for tensor maximum shear strain. Across nine FGM cases pooled over both sides (*n* = 18 model-sides), the corresponding factors were 3.51 ± 2.17 and 5.69 ± 4.03. In the fully matched homogeneous control (*D*/*R* = 1.0; DC/RC = 7.0/7.0 MPa) and the thickness-matched FGM control (*D*/*R* = 1.0), concentration factors were approximately 1. In contrast, every thickness-mismatched model-side had K > 1 for both strain measures, indicating elevation of local-ROI 95th percentile strain relative to that model-side’s far-field median strain.

**Table 3:** Local/far-field 95th percentile strain concentration factors at the donor-recipient interface

Model family	Pooled analysis set	*n*	${K}_{95}$, compressive strain Mean ± SD	${K}_{95}$, maximum shear strain Mean ± SD
Homogeneous linear elastic	99 configurations × 2 sides	198	3.71 ± 2.04	6.03 ± 3.79
FGM	9 ratios × 2 sides	18	3.51 ± 2.17	5.69 ± 4.03

Values are mean ± one standard deviation across side-specific model regions. The homogeneous sample comprises 99 configurations × 2 sides (*n* = 198), and the FGM sample comprises nine ratios × 2 sides (*n* = 18). ${K}_{95}$ was calculated as the local-interface-ROI 95th percentile strain divided by the same-side far-field median strain. The local interface ROI was defined as $0<\left|x-8\right|\le 0.25$ mm from the donor-recipient interface, and the far-field band was defined as $0.75\le \left|x-8\right|\le 1.00$ mm. A value greater than 1 indicates local interface strain amplification relative to each model’s own far-field strain environment

### Effect of depth-dependent material behavior on strain

**Fig. 6:**
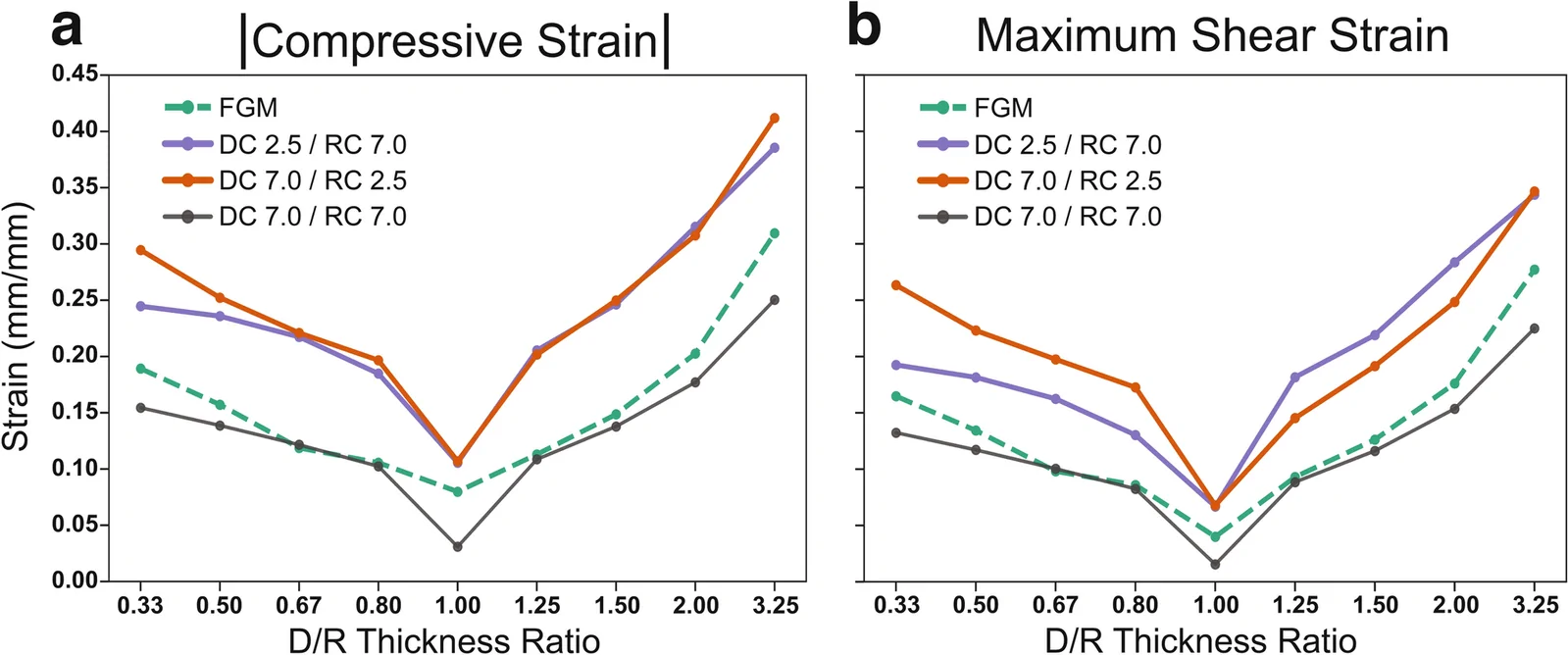
Local-interface-ROI 95th percentile comparison of depth-dependent FGM and homogeneous material assignments. The larger side-specific 95th percentile value for **a** compressive strain magnitude and **b** tensor maximum shear strain is plotted against *D*/*R* ratio. FGM models are compared with homogeneous DC/RC = 2.5/7.0, 7.0/2.5, and 7.0/7.0 MPa references. FGM values were lower than both extreme homogeneous mismatch references at every evaluated *D*/*R* ratio. FGM reduced local strains relative to the most extreme homogeneous mismatch cases across the thickness-ratio range but did not eliminate interface-local strain amplification

Local-interface-ROI 95th percentile compressive and maximum shear strain values for the FGM models are shown in Fig. 6 and compared with the extreme homogeneous modulus-mismatch reference cases (DC/RC = 2.5/7.0 and 7.0/2.5 MPa) and the homogeneous matched control (DC/RC = 7.0/7.0 MPa). In the thickness-matched FGM case (*D*/*R* = 1), local 95th percentile compressive strain was 0.080, exceeding the homogeneous matched-control value of 0.031 but remaining below the extreme homogeneous mismatch cases. Within both the *D*/*R* > 1 and *D*/*R* < 1 branches, both strain outcomes increased monotonically as the magnitude of thickness disparity increased. At *D*/*R* = 3.25, local 95th percentile compressive strain increased to 0.309, representing an approximately 286% increase relative to the thickness-matched FGM case. At *D*/*R* = 0.33, it increased to 0.189, representing an approximately 136% increase (Fig. 6a). Maximum shear strain followed a similar pattern. The thickness-matched FGM case had a local 95th percentile maximum shear strain of 0.040, whereas values increased to 0.277 at *D*/*R* = 3.25 and 0.165 at *D*/*R* = 0.33, corresponding to increases of approximately 593% and 312%, respectively (Fig. 6b). Across all thickness ratios, the FGM cases produced lower local 95th percentile strain than the extreme homogeneous modulus-mismatch references, although interface-local strain remained elevated at the largest thickness disparities.

## Discussion

This study used simplified two-dimensional FE models to quantify how mismatches between donor-to-recipient cartilage thickness and mechanical property alter the local strain environment at the graft boundary of a patellar OCA transplant. Increasing departure from matched thickness was associated with higher local-interface-ROI 95th percentile compressive strain magnitude and tensor maximum shear strain. Concentration factors were greater than 1 in all thickness-mismatched model-sides, indicating upper-tail elevation relative to the same model’s same-side far field; matched homogeneous and FGM controls were approximately 1. Together, these findings show that interface-local strain depended on thickness geometry and the distribution of stiffness across the donor and recipient regions.

The increase in the observed strain is interpreted as a thickness-driven strain concentration mechanism that is caused by the discontinuity in the cartilage thickness between donor and recipient sections and the change of stiffness across the donor-recipient interface. In the matched- thickness models (*D*/*R* = 1), strain values are low or negligible, and they are distributed smoothly throughout the cartilage. In contrast, introducing a thickness or stiffness mismatch in the model generated a local constraint that redirects deformation toward the interface, resulting in the local HSR being observed. At matched thickness, the two extreme reciprocal modulus-mismatch cases (DC/RC = 2.5/7.0 and 7.0/2.5 MPa) produced higher local strain than the 7.0/7.0 MPa control. Additional matched-modulus simulations showed that lower absolute modulus could elevate local 95th percentile strain under the prescribed pressure even without a donor-recipient modulus discontinuity (Supplemental Information, Appendix D). While lower matched-modulus simulations increased strain at the interface, the presence of stiffness mismatch models still resulted in higher strains at the donor-recipient interface, particularly when stiffness differences coincided with geometric mismatch. This is consistent with prior experimental and computational studies demonstrating that shear deformation in articular cartilage is sensitive to surface integrity, collagen organization, and material heterogeneity (Maier et al. 2017; Trevino et al. 2017).

In the matched-stiffness homogeneous models (DC/RC = 7.0/7.0 MPa), departure from matched cartilage thickness was associated with higher local 95th percentile compressive and maximum shear strain. Donor-side-thicker configurations (*D*/*R* > 1) produced higher values than their paired recipient-side-thicker configurations (*D*/*R* < 1), with the difference narrowing as *D*/*R* approached 1. The present strain-based analysis extends the work done by Rosario et al. (Rosario et al. 2023) by examining a donor/recipient modulus matrix and depth-dependent FGM cases and by combining local-ROI 95th percentile outcomes with same-model far-field normalization. Because recipient thickness was fixed at 2 mm, opposing *D*/*R* configurations were not geometrically equivalent: Donor-thicker models had larger absolute thickness differences than the corresponding recipient-thicker models. Consequently, their higher interface-local strains may reflect both mismatch direction and thickness-step magnitude, which were not independently varied in the present design.

Incorporating depth-dependent cartilage stiffness through the FGM model definition reduced local 95th percentile strain magnitudes relative to both extreme homogeneous modulus-mismatch references at every *D*/*R* ratio, and the pooled mean concentration factors were descriptively lower. The FGM formulation, however, did not eliminate the interface-local strain elevation associated with cartilage thickness disparity. In the thickness-matched FGM model (*D*/*R* = 1), the side-specific concentration factors were approximately 1, indicating that its higher absolute strain relative to the homogeneous 7.0/7.0 MPa control was not concentrated at the donor-recipient interface relative to the FGM model’s own far field. In contrast, all thickness-mismatched FGM models had concentration factors greater than 1, and local-ROI 95th percentile compressive and maximum shear strains increased as *D*/*R* departed from 1. This persistence suggests that mismatch-associated localization was not solely an artifact of homogeneous material assumptions and is consistent with a contribution from donor-recipient geometry, although the present design does not isolate that contribution. Because the model families were not matched for their through-thickness or effective stiffness distributions, the lower FGM values cannot be attributed to grading alone. Together, these findings indicate that depth-dependent material behavior modified the predicted strain magnitudes but did not remove the association between cartilage thickness mismatch and interface-local strain elevation.

Experimental studies have associated elevated compressive and shear strains have been associated with chondrocyte injury (Bartell et al. 2015), apoptosis (Thomas et al. 2011), and matrix degradation (Trevino et al. 2017) in articular cartilage. The sensitivity of shear strain values to cartilage thickness disparity is especially important, as shear deformation has been noted as a driver of cartilage damage and cell death (Trevino et al. 2017; Ayala et al. 2021). Within this framework, the HSR predicted in this study motivates testing whether donor-recipient mismatch creates a distinct mechanical and biological environment near the graft rim; it does not by itself demonstrate injury. Recent displacement-encoded magnetic resonance imaging of patellar OCA specimens demonstrated the feasibility of measuring full-volume cartilage strain and observed sample-specific redistribution near portions of the graft boundary (Hernández Lamberty et al. 2026). Those observations motivate experimental studies focused on interface heterogeneity, but they do not validate the present strain magnitudes, diagnostic thresholds, or behavior under in vivo joint loading.

The axisymmetric geometry, fixed subchondral boundary, spatially uniform pressure, and idealized interface omit patient-specific curvature, flexion-dependent contact, graft seating, and joint kinematics. The 20 µm radius transition regularized a nonphysiological sharp corner but does not represent measured graft-edge morphology; therefore, the magnitude and distribution of steep local gradients remain dependent on the assumed transition geometry. Excluding exact interface nodes and using a local 95th percentile reduced dependence on a single numerical extremum, while mesh and geometry sensitivity analyses supported numerical robustness; these steps do not make the absolute strain values patient-specific. An initial-stiffness-matched neo-Hookean subset produced modest but direction-dependent changes in local-ROI 95th percentile relative to exact linear references; it therefore serves only as a constitutive sensitivity analysis and not as validation of nonlinear cartilage behavior (Supplemental Information, Appendix E). Homogeneous and depth-dependent linear elasticity omit fluid pressurization, strain-dependent permeability, collagen fiber anisotropy, tension–compression nonlinearity, and viscoelastic time dependence known to influence cartilage mechanics (Soltz and Ateshian 1998; Huang et al. 2001; Maier et al. 2017; Lawless et al. 2017). Some modeled strains extend beyond the range in which linear elasticity provides a realistic constitutive description; particularly large magnitudes should therefore be interpreted comparatively rather than as quantitative tissue-level predictions. Finally, the 0.18 HSR threshold is a diagnostic analysis choice and the 95th percentile and concentration factors are model-derived descriptors rather than injury criteria.

In summary, increasing donor-recipient cartilage thickness disparity in this idealized model was consistently associated with greater local-ROI 95th percentile compressive and maximum shear strain at the patellar OCA interface. Concentration factors greater than 1 in mismatched cases indicated that these elevations were localized relative to each model’s same-side far-field response. Stiffness magnitude and donor-recipient stiffness contrast also modified the local response, while the FGM models produced lower local 95th percentile strains than the extreme homogeneous modulus-mismatch references at every *D*/*R* ratio but retained mismatch-associated localization. These findings support evaluating cartilage thickness and mechanical compatibility alongside surface congruity and osseous integration in future experimental and patient-specific modeling studies. They do not establish graft-selection tolerances, predict integration or failure, or define an injury threshold.

## Supplementary Information

Below is the link to the electronic supplementary material.

Supplementary file1 (DOCX 941 kb)

## Data Availability

The finite element model files and derived data presented in this study are available from the corresponding author upon reasonable request.
