# Supplementary material for: Combined cartilage thickness and mechanical property mismatch drives local strain amplification at the patellar osteochondral allograft interface
Source: Biomech Model Mechanobiol. 2026 Sep 12;25(5):102. doi: 10.1007/s10237-026-02128-9 (PMC13570921; doi:10.1007/s10237-026-02128-9)
Supplement: Supplementary file 1 [file 10237_2026_2128_MOESM1_ESM.docx]

Supplemental Materials

# Governing analytical convention and notation

D/R is donor-cartilage thickness divided by recipient-cartilage thickness. DC/RC denotes the ordered donor-cartilage/recipient-cartilage Young's modulus assignment. P95 is the 95^th^ percentile of nodewise strain within the stated analysis region. HSR denotes a threshold-defined high strain region; it is a numerical containment diagnostic, not a biological injury region. FGM denotes the functionally graded material formulation.

Tensor-first governs every strain result. Abaqus element-nodal ${LE}_{11}$, ${LE}_{22}$, ${LE}_{33}$, and engineering ${LE}_{12}$ contributions were averaged componentwise at each node and separately within donor and recipient element sets. The tensor component was $\varepsilon_{12}=\frac{{LE}_{12}}{2}$. The three-dimensional extrema $\varepsilon_{min}$ and $\varepsilon_{max}$ included ${LE}_{33}$. Compressive strain magnitude was max(0, ${-\varepsilon}_{min}$), and tensor maximum shear strain was $(\frac{\varepsilon_{max}-\varepsilon_{min}}{2})$. Exact x = 8 mm interface nodes were excluded before percentile calculations unless a source-audit exception is explicitly identified.

For consistency with the main manuscript, K denotes local-ROI P95 divided by the same model's same-side far-field median. The local ROI is 0 < |x − 8 mm| ≤ 0.25 mm, and the far-field band is 0.75 ≤ |x − 8 mm| ≤ 1.00 mm. Concentration-factor results remain in the main manuscript and are not duplicated in these supplements.

Appendix A: Whole-model deformation and boundary-buffer quality assurance

# A1. Validation objective

To document the applied supports and loading and inspect whole-model deformation and vertical-displacement patterns in representative recipient-side-thicker, thickness-matched, and donor-side-thicker configurations. This check addresses whether the fixed subchondral boundary and unloaded outer-edge buffer produce gross deformation artifacts that encroach on the donor-recipient interface region.

# A2. Whole-model assessment

The finalized 3 × 3 Abaqus composite was inspected for D/R = 0.33, 1.00, and 3.25. Columns correspond to thickness ratio; rows show boundary/load markers, deformed shape, and vertical displacement U2. Translation was fixed along the subchondral surface, axisymmetry was applied at the central axis, and a spatially uniform nominal pressure of 1.00 MPa was applied over a 26 mm radius, leaving a 2 mm unloaded outer-edge buffer. The primary donor and recipient regions share a conforming continuous mesh. Images were evaluated qualitatively at the final compression frame; no contour value was digitized.

# A3. Deformation and boundary-buffer findings

Support and load markers are present in all three configurations and are consistent with the prescribed boundary conditions. Deformed outlines remain continuous through the donor-recipient junction without visible rigid-body motion or a gross displacement discontinuity. U2 varies smoothly across the model span, and changes near the thickness step are spatially separated from the outer loaded-to-unloaded transition (**Fig. A1**).

# A4. Interpretation

The composite supports the intended global kinematics across the tested thickness range and shows that the interface region is not visually conflated with the outer loading edge. This is qualitative corroboration that the interface-local response is not simply the outer-edge displacement pattern; it is not proof of zero boundary influence.

**Fig A1:** Whole-model deformation and boundary-buffer views. Columns show D/R = 0.33, 1.00, and 3.25. Panels **a-c** show geometry with boundary-condition and surface-load markers; panels **d-f** show deformed shapes; panels **g-i** show U2 at the final compression frame. Translation was fixed along the subchondral surface, axisymmetry was applied at the central axis, and uniform nominal pressure was applied over a 26 mm radius with a 2 mm unloaded edge buffer. Contours are qualitative, use display-dependent scales, and have no error bars.

Appendix B: Local ROI and HSR-extent validation

# B1. Validation objective

To distinguish the fixed local region used to calculate P95 strain from the threshold-based HSR-extent analysis and to determine whether the HSRs identified at the 0.18 diagnostic threshold were contained within the 0.25-mm local ROI.

# B2. Fixed ROI and diagnostic HSR extent

The local ROI was fixed at $0<\left| x-8 \text{mm} \right|\leq0.25$ mm on each side of the donor-recipient interface and was applied consistently across all models. Side-specific P95 strain was calculated from all nodes within this fixed ROI. The HSR-extent analysis was performed separately as a containment check and did not determine the ROI width.

HSR extent was evaluated separately on the donor and recipient sides for compressive strain magnitude and tensor maximum shear strain. For each side and strain measure, nodes with strain greater than or equal to 0.18 were identified. When one or more nodes met this threshold, HSR extent was defined as the distance from the donor-recipient interface to the farthest threshold-exceeding node. When no node met the threshold, the extent was recorded as zero. The 0.18 threshold was applied to individual nodal strain values only for the HSR-extent analysis; the side-specific P95 strain was calculated separately and was not required to exceed 0.18.

The 0.18 threshold was used only to evaluate containment of the localized high-strain region and was not treated as a validated biological or clinical injury threshold. Because the donor and recipient sides were evaluated separately, the 99 homogeneous models provided 198 analyzed sides for each strain measure, and the nine FGM models provided 18 analyzed sides for each strain measure.

# B3. HSR-extent findings

At the 0.18 diagnostic threshold, at least one node met the threshold on 79 of the 198 analyzed homogeneous-model sides for compressive strain magnitude and on 53 sides for tensor maximum shear strain. The farthest threshold-exceeding node was 0.0218 mm from the interface for compression and 0.0138 mm from the interface for maximum shear.

Among the 18 analyzed FGM-model sides, at least one node met the threshold on 4 sides for compressive strain magnitude and on 3 sides for tensor maximum shear strain. The corresponding maximum extents were 0.0125 mm for compression and 0.0113 mm for maximum shear.

The largest HSR extent observed in either model family was 0.0218 mm, which was well within the local ROI boundary located 0.25 mm from the interface. Therefore, every HSR identified at the 0.18 diagnostic threshold was contained within the fixed local ROI (**Table B1**).

**Table B1:** Verified HSR-extent results at the 0.18 diagnostic threshold

| **Model family** | **Metric** | **Analyzed sides, n** | **With HSR, n** | **Maximum extent (mm)** | **Local ROI boundary from interface (mm)** |
| --- | --- | --- | --- | --- | --- |
| Homogeneous  Linear Elastic | Compressive strain magnitude | 198 | 79 | 0.021801 | 0.25 |
| Homogeneous  Linear Elastic | Tensor maximum shear strain | 198 | 53 | 0.013781 | 0.25 |
| FGM | Compressive strain magnitude | 18 | 4 | 0.012500 | 0.25 |
| FGM | Tensor maximum shear strain | 18 | 3 | 0.011250 | 0.25 |

Appendix C: Model, geometry, and FGM implementation validation

# C1. Validation scope

To document sensitivity to interface regularization and material-boundary assignment, verify continuous FGM implementation, and quantify local 0.25 mm ROI P95 sensitivity. Only models that reached the normalized step endpoint were included in numerical comparisons.

# C2. Chamfer, Fillet, and FilletHalf comparison methods

The submitted Chamfer, original circular Fillet, and current FilletHalf formulations were compared. The current FilletHalf geometry used a 20 µm fillet radius with 10 µm side dimensions on the donor and recipient sides of the material boundary at x = 8 mm. The term FilletHalf denotes the centered donor/recipient partition of the rounded transition and does not indicate that the fillet radius was halved. D/R = 1 has no thickness corner. Chamfer LE components were re-extracted from the ODBs, and all three methods were recomputed with the governing tensor-first Method A. P95 was calculated separately in the donor and recipient 0 < |x − 8 mm| ≤ 0.25 mm ROIs; each model was represented by the larger side-specific value for each metric. A model was eligible only if its final normalized frame value equaled 1.0 within ${10}^{-9}$. Pair-specific completed-case counts were 83 for Original Fillet versus Chamfer, 87 for FilletHalf versus Chamfer, and 83 for FilletHalf versus Original Fillet. Percent change was $\frac{\left( Candidate - Reference \right)}{Reference}\times100$.

Of 88 readable Chamfer and 88 Original Fillet source models, one Chamfer and five Original Fillet models stopped before the normalized step endpoint and were excluded; all 99 primary FilletHalf models reached the endpoint.

# C3. Fillet and FilletHalf FGM comparison methods

Original Fillet and FilletHalf FGM cases were paired at each of nine D/R ratios. All 18 source models reached the normalized step endpoint. Compressive strain magnitude and tensor maximum shear were recomputed from nodewise $LE$ components, summarized as side-specific local 0.25 mm ROI P95 values, and represented by the larger donor- or recipient-side P95. Percent change was $\frac{\left( FilletHalf - Fillet \right)}{Fillet}\times100$.

# C4. FGM Solution Dependent Variable (SDV) continuity and modulus definition

Donor and recipient cartilage used separate field-variable-dependent elastic materials. User-defined field (USDFLD) assigned FIELD(1) = COORD(2) = y at material integration points. For region r (DC or RC), Young's modulus was:

$$E_{r}\left( y_{i} \right)=E_{DZ}+\left[ \frac{E_{STZ}-E_{DZ}}{y_{STZ,r}-y_{DZ,r}} \right]\left( y_{i}-y_{DZ,r} \right)$$

Here $E_{DZ}$ = 7.0 MPa and $E_{STZ}$= 2.5 MPa. Coordinates were defined separately for donor and recipient cartilage in each geometry. SDV1 and SDV2 recorded donor and recipient modulus functions. One FilletHalf FGM ODB per D/R ratio (n = 9) was checked by fitting each SDV to Field Variable (FV) 1 at integration points.

# C5. Chamfer, Fillet, and FilletHalf findings

After incomplete models were removed, Original Fillet versus Chamfer local-ROI P95 changed across 83 pairs by a mean of +9.819% for compression and +12.844% for tensor maximum shear; median changes were +11.806% and +16.309%. FilletHalf versus Chamfer increased in all 87 completed pairs, with mean changes of +19.014% and +30.382% and median changes of +18.513% and +30.242%. Across 83 completed FilletHalf-versus-Original Fillet pairs, mean changes were +8.933% and +17.093% (**Table C1**; **Fig. C1**).

**Table C1:** **Full-step-only matched geometry-method local 0.25 mm ROI P95 comparisons**

| **Comparison and metric** | **n** | **Mean (%)** | **Median (%)** | **Minimum (%)** | **Maximum (%)** | **Increase/decrease** |
| --- | --- | --- | --- | --- | --- | --- |
| Original Fillet vs Chamfer — Compression | 83 | +9.819 | +11.806 | -7.513 | +21.045 | 75 / 8 |
| Original Fillet vs Chamfer — Tensor max shear | 83 | +12.844 | +16.309 | -15.804 | +34.551 | 72 / 11 |
| FilletHalf vs Chamfer — Compression | 87 | +19.014 | +18.513 | +7.588 | +28.327 | 87 / 0 |
| FilletHalf vs Chamfer — Tensor max shear | 87 | +30.382 | +30.242 | +8.708 | +42.124 | 87 / 0 |
| FilletHalf vs Original Fillet — Compression | 83 | +8.933 | +8.165 | -5.854 | +32.095 | 76 / 7 |
| FilletHalf vs Original Fillet — Tensor max shear | 83 | +17.093 | +13.597 | -6.762 | +65.325 | 77 / 6 |

**Fig C1**: Full-step-only geometry-method sensitivity of local 0.25 mm ROI P95. Panels show mean completed-pair percent changes relative to Chamfer for (a) compressive strain magnitude and (b) tensor maximum shear strain. Original Fillet versus Chamfer includes n = 83 completed pairs; FilletHalf versus Chamfer includes n = 87. Per-ratio means use the completed pairs available at that ratio. Lines are visual guides; no error bars are shown.

# C6. FGM geometry-comparison findings

All nine FGM pairs reached the full step. FilletHalf FGM local-ROI P95 values were equal to or greater than Original Fillet values at every D/R ratio. Across the nine pairs, mean changes were +2.544% for compression and +3.176% for tensor maximum shear. The largest compression change was +6.277% at D/R = 3.25; the largest shear change was +6.873% at D/R = 0.33. D/R = 1.00 and 0.67 were unchanged at the reported precision (**Table C2**; **Fig. C2**).

**Table C2:** **Full-step FGM FilletHalf-versus-Original Fillet local 0.25 mm ROI P95 changes**

| **D/R** | **Compression P95 change (%)** | **Tensor maximum-shear P95 change (%)** |
| --- | --- | --- |
| 0.33 | +5.050 | +6.873 |
| 0.50 | +3.185 | +5.031 |
| 0.67 | +0.000 | +0.000 |
| 0.80 | +1.369 | +1.744 |
| 1.00 | +0.000 | +0.000 |
| 1.25 | +1.231 | +1.756 |
| 1.50 | +1.966 | +3.122 |
| 2.00 | +3.815 | +4.533 |
| 3.25 | +6.277 | +5.528 |

**Fig C2:** Full-step FGM geometry-method sensitivity of local 0.25 mm ROI P95. Panels show FilletHalf-versus-Original Fillet percent changes for (a) compressive strain magnitude and (b) tensor maximum shear strain at each D/R ratio. One completed deterministic matched pair is shown per ratio. Lines are visual guides; no error bars are shown.

# C7. FGM implementation and SDV continuity findings

In the D/R = 3.25 check, 500,436 integration-point records contained 432,827 unique SDV1 values and 466,038 unique SDV2 values after rounding to six decimal places; maximum equation residuals were 4.91 × $10⁻⁷$and 1.15 × $10⁻⁶$. These results confirm a continuously evaluated integration-point field. Apparent discrete bands were contour-display intervals, not discrete materials (**Table C3**).

**Table C3:** Ratio-specific FGM diagnostic state-variable equations

| **D/R** | **SDV1 equation** | **SDV2 equation** |
| --- | --- | --- |
| 0.33 | SDV1 = -6.618y + 15.732 | SDV2 = -2.25y + 7 |
| 0.50 | SDV1 = -4.455y + 11.411 | SDV2 = -2.25y + 7 |
| 0.67 | SDV1 = -3.358y + 9.2164 | SDV2 = -2.25y + 7 |
| 0.80 | SDV1 = -2.795y + 8.0901 | SDV2 = -2.25y + 7 |
| 1.00 | SDV1 = -2.25y + 7 | SDV2 = -2.25y + 7 |
| 1.25 | SDV1 = -1.8y + 7 | SDV2 = -2.25y + 8.125 |
| 1.50 | SDV1 = -1.5y + 7 | SDV2 = -2.239y + 9.2164 |
| 2.00 | SDV1 = -1.125y + 7 | SDV2 = -2.239y + 11.455 |
| 3.25 | SDV1 = -0.6923y + 7 | SDV2 = -2.239y + 17.052 |

# C8. Interpretation

FilletHalf local-ROI P95 values were higher than Chamfer in all 87 completed matched cases. FilletHalf was higher than Original Fillet in 76 of 83 compression pairs and 77 of 83 tensor maximum-shear pairs, but not in every completed comparison. The FGM Fillet-versus-FilletHalf differences were comparatively modest, and continuous SDV fits rule out interpretation of the FGM as discrete layers.

Appendix D: Matched-modulus controls

# D1. Control objective

To distinguish strain associated with absolute cartilage compliance from strain associated with a donor-recipient modulus discontinuity.

# D2. Matched-control design and local-ROI comparison metric

Five matched assignments (DC/RC = 2.5/2.5, 3/3, 4/4, 5/5, and 6/6 MPa) were attempted at each of nine D/R ratios, yielding 45 attempted controls. Each completed control was paired with two stiffness-mismatch cases at the same soft modulus and D/R ratio: DC = 7 MPa with a softer RC, and a softer DC with RC = 7 MPa. P95 was calculated separately in the donor and recipient local ROIs, defined as 0 < |x − 8 mm| ≤ 0.25 mm, after excluding exact x = 8 mm nodes. For each strain metric, the larger donor- or recipient-side P95 represented the model. The comparison ratio was this case-level maximum-side P95 in the mismatch case divided by the corresponding value in the equally soft matched control. The donor or recipient side attaining the maximum was selected independently in the numerator and denominator.

$$R_{mismatch/matched, m}=\frac{\max_{s\in\left\{ D,R \right\}} \left( {P95}_{m,s}^{\mathrm{mismatch}} \right)}{\max_{s\in\left\{ D,R \right\}} \left( {P95}_{m,s}^{\mathrm{matched}} \right)}$$

# D3. Completion and mismatch-to-matched findings

Thirty-eight of 45 controls reached the final load state; seven failed (**Fig. D1**). Across 76 completed pairings, the median mismatch-to-matched local-ROI P95 ratio was 0.683 (interquartile range [IQR], 0.590–0.764; range, 0.466–1.237) for compression and 0.643 (IQR, 0.550–0.730; range, 0.411–1.570) for tensor maximum shear. All 66 ratios from thickness-mismatched geometries (D/R ≠ 1) were below 1 for both metrics. At D/R = 1, the median ratios were 1.083 for compression and 1.230 for tensor maximum shear; 8 of 10 compression ratios and all 10 maximum-shear ratios exceeded 1 (**Table D1**; **Fig. D2**).

**Fig D1.** Matched-control completion status. Five matched DC/RC assignments were attempted across nine D/R ratios (45 cases). Green C cells completed the final compression step; gray F cells failed and were excluded. Thirty-eight cases completed and seven failed. No error bars are applicable.

**Table D1:** Corrected mismatch-to-matched local-ROI P95 ratios by D/R

| **D/R** | **Ratio observations, n** | **Compression median (IQR)** | **Tensor maximum-shear median (IQR)** |
| --- | --- | --- | --- |
| 0.33 | 8 | 0.659 (0.578–0.729) | 0.634 (0.539–0.708) |
| 0.50 | 8 | 0.667 (0.592–0.728) | 0.634 (0.549–0.703) |
| 0.67 | 10 | 0.625 (0.543–0.704) | 0.587 (0.519–0.666) |
| 0.80 | 10 | 0.647 (0.568–0.724) | 0.614 (0.549–0.691) |
| 1.00 | 10 | 1.083 (1.020–1.178) | 1.230 (1.083–1.440) |
| 1.25 | 10 | 0.643 (0.568–0.717) | 0.605 (0.548–0.683) |
| 1.50 | 10 | 0.628 (0.545–0.707) | 0.590 (0.505–0.674) |
| 2.00 | 6 | 0.694 (0.637–0.745) | 0.663 (0.615–0.714) |
| 3.25 | 4 | 0.725 (0.686–0.760) | 0.707 (0.669–0.741) |

**Fig D2.** Mismatch-to-matched local-ROI P95 ratios. Case-level ratios are shown for compressive strain magnitude and tensor maximum shear across D/R ratios. Circles denote DC = 7 MPa/RC soft; squares denote DC soft/RC = 7 MPa. The dashed line denotes unity. There are 76 paired ratios from 38 completed controls. Points are deterministic cases; no error bars are shown

# D4. Interpretation

Under the prescribed nominal pressure, lower modulus on both sides increased interface-local deformation without a modulus discontinuity. Every completed thickness-mismatched soft/soft control had a larger case-level maximum-side P95 than its one-soft/one-7-MPa counterpart. At D/R = 1, stiffness contrast increased maximum shear in all comparisons and compression in most. Thus, absolute compliance and stiffness contrast both influenced local strain; the largest values cannot be attributed to modulus discontinuity alone.

**D5. Limitations**

Seven failed controls clustered in low-modulus, large-thickness-mismatch cells; no values were imputed. Because the larger side was selected independently, ratios are case-level rather than same-side causal comparisons. These idealized models do not provide clinical predictions.

**Appendix E: Neo-Hookean constitutive sensitivity**

# E1. Constitutive-sensitivity objective

To test whether replacing the linear-elastic response with a finite-strain Neo-Hookean form, while matching initial small-strain stiffness, materially changed local-ROI P95 to a selected subset.

# E2. Parameter conversion and paired-case methods

For the compressible Abaqus Neo-Hookean form $W=C_{10}\left( \bar{I}_{1}-3 \right)+\frac{\left( J-1 \right)^{2}}{D_{1}}$, the initial shear and bulk moduli are $G_{0}=2C_{10}$ and $K_{0}=\frac{2}{D_{1}}$. Matching Young's modulus E and Poisson's ratio ν gives:

$$C_{10}=\frac{E}{4\left( 1+\nu\right)}, D_{1}=\frac{6\left( 1-2\nu\right)}{E}$$

With ν = 0.46, the implied parameter pairs are $C_{10}$ = 0.4281 MPa and $D_{1}$ = 0.1920 MPa⁻¹ for E = 2.5 MPa, and $C_{10}$ = 1.1986 MPa and $D_{1}$ = 0.06857 MPa⁻¹ for E = 7.0 MPa. These values are analytical conversions from the stated linear inputs; the curated bundle omits the archived Neo-Hookean input deck, so direct deck verification remains necessary before publication (**Table E1**).

**Table E1:** Initial-stiffness-equivalent Neo-Hookean parameters

| **Linear E (MPa)** | **ν** | $\boldsymbol{C}_{\boldsymbol{10}}$ **(MPa)** | $\boldsymbol{D}_{\boldsymbol{1}}$ **(MPa⁻¹)** |
| --- | --- | --- | --- |
| 2.5 | 0.46 | 0.4281 | 0.1920 |
| 7.0 | 0.46 | 1.1986 | 0.06857 |

Nine Neo-Hookean jobs were completed: two at D/R = 1, three at D/R = 3.25, and four at D/R = 0.33. Eight had an exact linear reference with identical D/R and DC/RC assignment. The D/R = 0.33, DC/RC = 2.5/2.5 MPa case lacked a completed reference and was excluded from ratios. For each exact pair, P95 was calculated separately in the donor and recipient local ROIs, the larger side was retained, and the ratio was Neo-Hookean/linear.

# E3. Neo-Hookean sensitivity findings

Across eight exact pairs, Neo-Hookean/linear local-ROI P95 ratios ranged from 0.984 to 1.163 for compression and 0.966 to 1.191 for tensor maximum shear. Six of eight compression ratios and five of eight maximum-shear ratios exceeded 1. The largest increase occurred at D/R = 3.25, DC/RC = 2.5/7 MPa; the opposing 7/2.5 MPa assignment decreased, demonstrating a directional constitutive response (**Tables E2-E3**; **Fig. E1**).

**Table E2:** Neo-Hookean versus linear local-ROI P95: compressive strain

| **D/R** | **DC/RC (MPa/MPa)** | **Reference** | **Neo-Hookean** | **Linear** | **Ratio** |
| --- | --- | --- | --- | --- | --- |
| 1.00 | 2.5/2.5 | Exact | 0.0894 | 0.0865 | 1.034 |
| 1.00 | 7/7 | Exact | 0.0313 | 0.0309 | 1.012 |
| 3.25 | 7/2.5 | Exact | 0.4055 | 0.4119 | 0.984 |
| 3.25 | 2.5/7 | Exact | 0.4483 | 0.3856 | 1.163 |
| 3.25 | 7/7 | Exact | 0.2492 | 0.2504 | 0.995 |
| 0.33 | 2.5/2.5 | No exact completed reference | 0.4950 | — | — |
| 0.33 | 7/2.5 | Exact | 0.2992 | 0.2946 | 1.016 |
| 0.33 | 2.5/7 | Exact | 0.2479 | 0.2447 | 1.013 |
| 0.33 | 7/7 | Exact | 0.1565 | 0.1544 | 1.013 |

**Table E3:** Neo-Hookean versus linear local-ROI P95: tensor maximum shear

| **D/R** | **DC/RC (MPa/MPa)** | **Reference** | **Neo-Hookean** | **Linear** | **Ratio** |
| --- | --- | --- | --- | --- | --- |
| 1.00 | 2.5/2.5 | Exact | 0.0447 | 0.0432 | 1.034 |
| 1.00 | 7/7 | Exact | 0.0156 | 0.0154 | 1.012 |
| 3.25 | 7/2.5 | Exact | 0.3347 | 0.3466 | 0.966 |
| 3.25 | 2.5/7 | Exact | 0.4094 | 0.3437 | 1.191 |
| 3.25 | 7/7 | Exact | 0.2216 | 0.2250 | 0.985 |
| 0.33 | 2.5/2.5 | No exact completed reference | 0.4238 | — | — |
| 0.33 | 7/2.5 | Exact | 0.2667 | 0.2634 | 1.013 |
| 0.33 | 2.5/7 | Exact | 0.1924 | 0.1925 | 0.999 |
| 0.33 | 7/7 | Exact | 0.1326 | 0.1324 | 1.002 |

**Fig E1.** Neo-Hookean/linear local-ROI P95 ratios. Bars show the larger side-specific local-ROI P95 ratio for compression and tensor maximum shear in eight exact-reference pairs. Modulus labels use manuscript-standard DC/RC order. The dashed line denotes unity. The D/R = 0.33, DC/RC = 2.5/2.5 MPa model is omitted because no exact linear reference completed. Bars represent deterministic cases; no error bars are shown.

**E4. Interpretation**

The finite-strain replacement produced mostly modest, case-dependent changes and retained the qualitative interface-local pattern. Opposing changes in the two D/R = 3.25 mismatch directions show that the effect was not a uniform scale factor. These results represent constitutive sensitivity within the tested formulation, not validation of absolute strain or all nonlinear cartilage behavior.

**E5. Limitations**

This analysis comprised a nine-case subset, and one case lacked an exact completed linear reference. The one-term isotropic Neo-Hookean model omitted fluid pressurization, permeability, collagen-fiber anisotropy, tension-compression asymmetry, and viscoelasticity. Initial-stiffness matching did not make the linear and Neo-Hookean responses equivalent at finite strain, and the parameter conversion should be confirmed against the archived input deck before publication.

Appendix F: Contact and friction sensitivity

# F1. Sensitivity objective

To evaluate two distinct interface assumptions: low-friction contact between donor and recipient cartilage at matched thickness, and friction at a bone-cartilage interface in separated-body FGM models.

# F2. Contact-model boundary and interaction formulation

Contact-enabled models required the donor and recipient cartilage to be represented as separate deformable bodies. In the primary continuous models, the subchondral surface could be constrained directly because the donor and recipient regions shared a conforming mesh. Once the cartilage regions were separated, particularly for thickness-mismatched geometries with D/R ≠ 1, the original shared-node boundary-condition formulation could not be applied consistently to both bodies while retaining the intended contact kinematics. An idealized rigid body representing the underlying bone was therefore introduced to provide a common subchondral support. Surface-to-surface interactions were defined between the donor and recipient cartilage surfaces and between each cartilage body and the rigid bone surface. This formulation permitted contact-pressure transfer and, where allowed by the specified interaction properties, relative sliding or separation between the contacting surfaces.

# F3. D/R=1 cartilage-cartilage contact methods

Three D/R = 1 FilletHalf separate-body cartilage-cartilage contact models used µ = 0.01 and DC/RC = 7/2.5, 2.5/7, or 7/7 MPa. Each was compared with the matching primary conforming continuous shared-node model. P95 was calculated separately on each side in the 0 < |x − 8 mm| ≤ 0.25 mm local ROI after excluding x = 8 mm; the larger side-specific P95 was compared as the contact/continuous ratio.

# F4. FGM bone-cartilage friction methods

Separated-body FGM jobs were evaluated at D/R = 3.25 with Bone-DC friction and D/R = 0.33 with Bone-RC friction. For each geometry, the completed friction sweep comprised µ = 0.25, 0.50, and 0.75. A D/R = 1 job with no active contact was retained as a topology reference but did not enter the friction comparison. Four low-friction jobs (µ = 0.05 and 0.10 in each mismatched geometry) ended before the final load and were excluded without interpolation.

For the six completed friction cases, final-frame strain components were re-extracted from the ODB files and processed with tensor-first. Donor and recipient cartilage were evaluated separately. The local ROI was 0 < |x − 8 mm| ≤ 0.25 mm, and exact x = 8 mm nodes were removed before spatial P95 calculation. The side with the larger P95 was identified independently for each metric rather than assumed in advance.

At D/R = 3.25, the requested path coordinate was y = 4.500 mm; 67 local-ROI nodes were retained on each cartilage side, and the mesh-connected donor path governed both outcomes. At D/R = 0.33, the donor path retained 67 nodes. The recipient path was reconstructed as a monotonic, mesh-connected route nearest y = 5.834 mm because the previous exact-coordinate selector did not provide a spatially resolved ROI; 107 recipient nodes were retained, with maximum absolute deviation of 0.00512 mm from the requested coordinate. All six analyzed models reached the final load.

# F5. D/R=1 cartilage-cartilage contact findings

All three D/R=1 contact cases completed. In the two stiffness-mismatched cases, compression P95 was 0.783-0.786 of the continuous reference and tensor maximum-shear P95 was 0.622-0.627. In the matched 7/7 MPa case, ratios were 1.031 and 1.043 (**Table F1**; **Fig. F1**).

**Table F1:** D/R=1 cartilage-cartilage contact sensitivity

| **DC/RC (MPa/MPa)** | **Compression contact** | **Compression continuous** | **Ratio** | **Tensor maximum shear contact** | **Tensor maximum shear continuous** | **Ratio** |
| --- | --- | --- | --- | --- | --- | --- |
| 7/2.5 | 0.0841 | 0.1070 | 0.786 | 0.0423 | 0.0679 | 0.622 |
| 2.5/7 | 0.0826 | 0.1056 | 0.783 | 0.0419 | 0.0668 | 0.627 |
| 7/7 | 0.0318 | 0.0309 | 1.031 | 0.0161 | 0.0154 | 1.043 |

**Fig F1.** D/R=1 cartilage-cartilage contact sensitivity. Bars show contact/continuous shared-node local-ROI P95 for compression and tensor maximum shear at µ = 0.01. Modulus labels use DC/RC order. The dashed line denotes unity. Three deterministic cases are shown; no error bars are applicable.

# F6. FGM bone-cartilage friction findings

The D/R = 1 no-contact topology reference and all six friction-sweep jobs at µ = 0.25–0.75 reached the final load. For D/R = 3.25, DC governed compression and tensor maximum shear at every completed friction level. Relative to µ = 0.25, compression P95 increased by 54.6% at µ = 0.50 and 92.0% at µ = 0.75; maximum-shear P95 increased by 67.3% and 113.2%, respectively. For D/R = 0.33, RC governed both metrics. Compression P95 increased by 32.8% and 44.7%, and maximum-shear P95 increased by 40.7% and 54.8%, at µ = 0.50 and 0.75, respectively, relative to µ = 0.25 (**Table F2**).

**Table F2:** Corrected FGM bone-cartilage friction local-ROI P95

| **D/R** | **µ** | **Governing side** | **n** | **Compression P95** | **Change vs µ = 0.25** | **Tensor maximum-shear P95** | **Change vs µ = 0.25** |
| --- | --- | --- | --- | --- | --- | --- | --- |
| 3.25 | 0.25 | DC | 67 | 0.1281 | 0.0% | 0.0999 | 0.0% |
| 3.25 | 0.50 | DC | 67 | 0.1980 | +54.6% | 0.1672 | +67.3% |
| 3.25 | 0.75 | DC | 67 | 0.2459 | +92.0% | 0.2130 | +113.2% |
| 0.33 | 0.25 | RC | 107 | 0.1116 | 0.0% | 0.0842 | 0.0% |
| 0.33 | 0.50 | RC | 107 | 0.1482 | +32.8% | 0.1184 | +40.7% |
| 0.33 | 0.75 | RC | 107 | 0.1615 | +44.7% | 0.1303 | +54.8% |

**Note.** Values are dimensionless tensor-first spatial P95 within 0 < |x − 8 mm| ≤ 0.25 mm after exclusion of exact-interface nodes. Bone-DC contact was varied for D/R = 3.25 and Bone-RC contact for D/R = 0.33. Donor and recipient cartilage were evaluated separately; the listed side had the larger P95 for both metrics, and n is the retained node count on that side. Changes are relative to µ = 0.25 within the same geometry. Each row is one completed deterministic model; no error bars are applicable.

# F7. Interpretation

The D/R=1 subset shows that allowing low-friction cartilage contact can substantially reduce interface-local upper-tail shear in stiffness-mismatched, equal-thickness models while minimally changing the matched case. These results support describing the continuous shared-node interface as an idealized integrated condition whose load transfer is interface-assumption dependent. This subset does not establish press-fit behavior across thickness mismatch.

In the six completed separated-body FGM cases, increasing µ from 0.25 to 0.75 monotonically increased governing local-ROI compression and tensor maximum-shear P95, more at D/R = 3.25 than at D/R = 0.33. DC governed at D/R = 3.25 and RC at D/R = 0.33, confirming that both sides must be assessed. Thus, bone-cartilage friction was influential in these geometries, but these comparisons varied only the bone-cartilage friction coefficient and do not isolate donor-recipient contact effects or define biological thresholds.

**F8. Limitations**

D/R=1 used three equal-thickness cases and assumed µ = 0.01. The FGM sweep used two mismatch geometries and three completed friction levels per geometry; four lower-friction jobs were partial, so no values were imputed or extrapolated below µ = 0.25. The D/R = 0.33 recipient path approximated y = 5.834 mm. Introducing contact required both separation of the cartilage bodies and replacement of the directly constrained cartilage base with a rigid bone support and surface interactions. Contact-to-continuous comparisons therefore reflect the combined effects of the interface formulation and modified support architecture; friction-coefficient effects were isolated only among models using the same separated-body rigid-bone architecture. These deterministic results are idealized, not patient-specific or clinically predictive.
